# The State of Intraoperative OCT in Vitreoretinal Surgery: Recent Advances and Future Challenges

**DOI:** 10.3390/tomography9050132

**Published:** 2023-09-01

**Authors:** Nicolò Ciarmatori, Marco Pellegrini, Francesco Nasini, Pietro Maria Talli, Laura Sarti, Marco Mura

**Affiliations:** 1 St. Anna University Hospital, University of Ferrara, 30010 Ferrara, Italy; crmncl1@unife.it (N.C.);; 2 Istituto Internazionale per la Ricerca e Formazione in Oftalmologia (IRFO), 47122 Forlì, Italy; 3 Ospedali Privati Forlì “Villa Igea”, Department of Ophthalmology, 47122 Forlì, Italy; 4 King Khaled Eye Specialist Hospital, Riyadh 11462, Saudi Arabia

**Keywords:** intraoperative OCT, vitreoretinal surgery, epiretinal membrane, macular hole, retinal detachment, subretinal surgery

## Abstract

Since its first introduction more than 30 years ago, optical coherence tomography (OCT) has revolutionized ophthalmology practice, providing a non-invasive in vivo cross-sectional view of the structures of the eye. Mostly employed in the clinical setting due to its tabletop configuration requiring an upright patient positioning, the recent advent of microscope-integrated systems now allows ophthalmologists to perform real-time intraoperative OCT (iOCT) during vitreoretinal surgical procedures. Numerous studies described various applications of this tool, such as offering surgeons feedback on tissue–instrument interactions in membrane peeling, providing structural images in macular hole repair, and showing residual subretinal fluid or perfluorocarbon in retinal detachment surgery. This narrative review aims at describing the state of the art of iOCT in vitreoretinal procedures, highlighting its modern role and applications in posterior segment surgery, its current limitations, and the future perspectives that may improve the widespread adoption of this technology.

## 1. Introduction

Since the first images were first published in 1993 by Fercher et al. [1], optical coherence tomography (OCT) has shifted from a research tool to an indispensable device that has revolutionized modern ophthalmology practice. By employing the principle of low-coherence interferometry, OCT provides non-invasive in vivo cross-sectional images of the structures of the eye. Although commonly employed in clinical settings due to its tabletop system requiring an upright patient positioning, the recent advent of hand-held and microscope-integrated systems has allowed ophthalmologists to perform real-time intraoperative OCT (iOCT) during anterior and posterior segment surgery. The detailed “real-time” display of the vitreoretinal interface and retinal layers during surgery provides surgeons with valuable feedback that may improve surgical techniques and outcomes [2]. To date, many reports in the literature have described possible surgical scenarios where iOCT could contribute to surgical decision making. However, it remains debated whether the contribution brought by iOCT justifies its routine and widespread adoption. This narrative review aims to highlight the most recent advances in this technology, reporting current applications of iOCT in vitreoretinal surgery, its impact on surgical techniques and outcomes, and the current limitations to the widespread use of this technology in routine clinical practice.

## 2. Methods

An extensive literature search was conducted on PubMed using keywords including: “optical coherence tomography”, “vitreoretinal surgery”, “intraoperative optical coherence tomography”, “pars-plana vitrectomy”, “intraoperative retinal imaging”, “microscope-integrated optical coherence tomography”, and all relevant synonyms and abbreviations. Moreover, references of selected papers were scanned manually to identify additional studies. The retrieved titles and abstracts were included for full-text review by two of the co-authors (N.C. and P.M.T.) if they reported relevant technical details, applications, or outcomes of iOCT.

## 3. Intraoperative OCT Devices

The first iOCT platforms described in the literature were portable machines such as the Bioptigen SDOIS/Envisu (Bioptigen, Research Triangle Park, Morrisville, NC, USA) and the Optovue IVue 100 (Optovue, Fremont, CA, USA). The former employed a hand-held OCT scan head that could potentially be mounted on a surgical microscope to provide 32.000 A-scans per second, producing high-density raster scans up to 1000 × 1000 with an axial detail down to 2.4 µm [3]. The latter consisted of a stand-mounted device that applied a beam wavelength of 830–850 nm to acquire up to 25.000 A-scans per second with an optical resolution of 5 µm [4]. The main advantage of these devices was their portability, which allowed their use in any operating room for a limited investment, with no need to change the microscope in use. However, the need to pause surgery to acquire the scans and the high susceptibility to motion artifacts significantly impacted surgical times, image quality, and reproducibility.

These inherent technical limitations led several independent groups to develop microscope-mounted systems like the ones described by Binder et al. in 2010 [5] and Ehlers and Tao in 2011 [6]. Briefly, an OCT unit was mounted on the surgical microscope to interface optically and mechanically with the microscope itself. This technical upgrade allowed the OCT scanner to share the optical path, the focal plane, and the foot pedal controls of the surgical microscope, making the process of image acquisition quicker and more efficient, overcoming some of the limitations of hand-held devices. However, this design was still unable to provide real-time imaging of the surgical maneuver, with a 6.6 min median time of paused surgery according to the results of the PIONEER Study [7]. Furthermore, microscope-mounted iOCT still relied on an extended optical system, bringing with it inherent loss of signal power and the development of optical aberrations.

In order to enable effective real-time feedback during surgery to successfully integrate OCT intraoperatively, microscope-integrated devices were developed. Compared to hand-held and microscope-mounted OCT machines working on a separate axis and focusing from the surgical microscope, microscope-integrated iOCT machines are built within the microscope and operate parfocal and coaxial to its optical path, with minimal alteration of the surgeon’s workflow.

**Figure 1:**
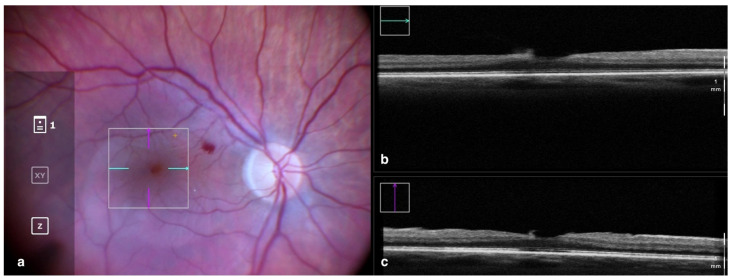
Rescan 700 iOCT showing on the left the en face ophthalmoscopic surgical view after staining and peeling. The digital overlay indicates the scanning area (**a**), and the horizontal and vertical scans are found in the upper and lower right portion of the image, respectively (**b**,**c**). The iOCT shows the surgeon the release of traction on the fovea and the complete removal of the ERM in the scanned area. Abbreviations: ERM, epiretinal membrane; iOCT, intraoperative optical coherence tomography.

At the time of writing, three models are commercially available (Table 1). The first model to be approved by the FDA and used on humans was the RESCAN 700 (Carl Zeiss Meditec, Oberkochen, Germany), a spectral-domain OCT system integrated into the OPMI Lumera 700 microscope platform and equipped with an external heads-up display (Figure 1).The OCT head of the RESCAN 700 can acquire 27.000 A-scans per second with an 840 nm wavelength, reaching 2 mm in depth with an axial resolution of 5.5 µm. Thanks to its ability to analyze a scan length of 3–16 mm and the possibility to rotate 360°, the RESCAN 700 can acquire images extending from the posterior pole to the equator, with an even wider field of view in selected cases with wide pupils and clear media [8,9]. After the Zeiss model, Leica Microsystems developed the EnFocus Ultra-HD intraoperative OCT (Leica Microsystems, Leica, Wetzlar, Germany), built into the Proveo 8 ophthalmic microscope. Its real-time display provides a 30 fps image to grant the surgeon an immediate feedback at each step, with 32,000 A-scans per second that can reach a depth of 2.5 mm with an axial resolution of 4 µm and a wavelength of 860 nm [10]. In both of these devices, the OCT beam is folded into the path of one of the oculars using a dichroic mirror placed and magnified prior to the microscope objective, with spherical relays placed to increase optical transmission. Lastly, Haag Streit commercialized an iOCT system (Haag Streit Surgical, Wedel, Germany), integrated into the HS Hi-R NEO 900A NIR microscope and characterized by a depth of view of 4.2 mm thanks to its wavelength of 840 nm and its axial resolution of 5 µm, with the ability to perform 10,000 A-scans per second. Similar in design to the previously described machines, Haag Streit’s iOCT utilizes a dichroic mirror to couple the OCT beam onto the optical path of the microscope which is placed after the microscope objective without the need for modification of the optics [11].

### 3.1. Clinical Applications in Vitreoretinal Surgery: Epiretinal Membrane

Epiretinal membranes (ERM) are among the most common indications for pars plana vitrectomy worldwide and one of the diseases best characterized using OCT imaging [12]. The gold standard of treatment consists of vitrectomy and peeling of the tissue causing distortion of the retina in order to relieve traction and restore the foveal structure and function. The information acquired with iOCT provides surgeons with helpful feedback on both the anatomical position in which to initiate the ERM peeling and on whether the membrane has been removed completely [13,14].

The benefit of iOCT for vitreoretinal surgery has been recently described in the PIONEER (Prospective Intraoperative and Perioperative Ophthalmic Imaging with Optical Coherence Tomography) paper, a large 2-year prospective study conducted by Ehlers and colleagues at the Cleveland Clinic including eyes with various posterior segment diseases treated surgically with the aid of iOCT imaging. The authors employed the Bioptigen SDOIS portable spectral-domain OCT system (Bioptigen, Research Triangle Park, NC, USA) mounted on either a Leica or Zeiss microscope with a custom-designed coupling mechanism compatible with BIOM^®^ as well as contact lens viewing systems. The study reported that in almost half of the procedures, iOCT had impacted the surgeon’s understanding of the surgical configuration and/or procedure, reversing surgical decision making in 13% of cases by revealing residual membranes that escaped the surgeon’s observation [7]. The DISCOVERY (Determination of Feasibility of Intraoperative Spectral Domain Microscope Combined/Integrated OCT Visualization During En Face Retinal and Ophthalmic Surgery) study was another prospective study from the same scientific group including patients who underwent vitreoretinal surgery and iOCT imaging with either the Zeiss Rescan 700, the Leica EnFocus, or the Cole Eye iOCT system [15]. In the study, the percentage of unnoticed epiretinal tissue remnants was even higher, with iOCT showing incompleteness of the procedure in 20% of peeling attempts considered successful and, on the other side, preventing unnecessary maneuvers in 40% of cases by showing the surgeon the absence of any epiretinal tissue leftovers [15].

This significant disagreement between the ability of iOCT and the human eye in detecting and determining the completeness of the peeling was further confirmed by other authors. In 2015, Falkner-Radler et al. described residual fragments of ERM or internal limiting membrane (ILM) remnants in almost 90% of patients after the first ERM peeling attempt [16], whereas Leisser and colleagues reported that in 20 cases of ERM surgery, residual tissue was still noticeable with iOCT imaging in 40% of cases, leading to the need for additional peeling to further reduce foveal traction [17].

Moreover, as reported by Kumar et al., iOCT imaging proved particularly helpful for ERM surgery on highly myopic eyes. Indeed, ERM peeling may result in the development of macular holes in myopic eyes with foveoschisis or retinal thinning. Thus, the images provided by iOCT may highlight safer zones to initiate the peeling and help to decide when to end the surgery once the retinal traction is relieved [18,19].

The level of detail provided by iOCT can also help to clarify how the retinal architecture responds to mechanical stretching during peeling procedures, providing more insight into how iatrogenic-induced changes might impact anatomical and visual outcomes. On this matter, Weschta and colleagues recently reported a negative correlation between intraoperative retinal stretching and postoperative thinning of the fovea in eyes undergoing ERM surgery [20].

Furthermore, various studies analyzed the feasibility of using iOCT as an alternative to dyes to stain the ERM and ILM. Even though recently developed dyes, such as trypan blue-based ones, are considered relatively safe for a short-time application [21,22,23], the toxicity of chromo-vitrectomy dyes, especially triamcinolone acetonide and indocyanine-green, still remains an important issue in macular surgery, and avoiding their use might reduce surgical time, retinal toxicity, and the rate of dye-related complications such as subretinal migration [24] or dye-related optic nerve atrophy [25]. In a study by Falkner-Radler and colleagues, stain-free peeling was feasible in one-third of cases with the application of iOCT [16]. This percentage was even higher in a recent report by Leisser et al. [26], where the discretional ability of imaging reproducibly highlighted the ERM without the need for staining, allowing the surgeons to perform dye-free peeling in 63% of cases. However, both studies did not report any significant difference in visual and anatomical outcomes between eyes undergoing iOCT-assisted peeling and conventional surgery. Moreover, staining was still required to complete more complex cases and identify and peel the ILM [27].

Interestingly, the signal intensity of the ERM and ILM in iOCT imaging is increased when dyes such as indocyanine green are used, as described by Ehlers et al. This suggests the potential combined use of iOCT and dyes to facilitate ERM and ILM identification in complex cases [28].

### 3.2. Clinical Applications in Vitreoretinal Surgery: Macular Hole

Macular holes are defined as partial or full-thickness defects in the foveal layers [29,30]. Full-thickness macular holes may be idiopathic or secondary to blunt or surgical trauma, high myopia, anti-VEGF intravitreal injections for age-related macular degeneration, and other concurrent conditions [31].

**Figure 2:**
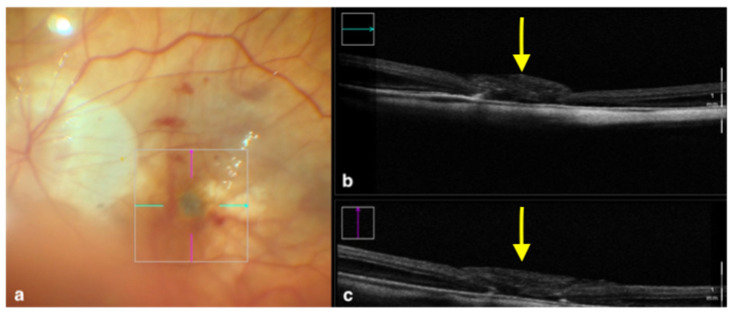
Rescan 700 iOCT showing an inverted ILM flap performed in a case of myopic full-thickness macular hole. The display simultaneously shows, on the left, the en face ophthalmoscopic surgical view with a digital overlay indicating the scanning area (**a**) and, in the upper and lower right portions of the image, the horizontal and vertical scans, respectively (**b**,**c**). The iOCT indicates to the surgeon the architecture of the hole and shows the positioning of the ILM flap (yellow arrows). Abbreviations: ILM, internal limiting membrane; iOCT, intraoperative optical coherence tomography.

The most common approach to treat full-thickness macular holes consists of vitrectomy and peeling of the ILM in order to release the centrifugal forces that caused the retinal tissue to split. For large or recurrent macular holes, further surgical measures are commonly employed in order to promote the anatomical closing process, such as filling the hole with inverted flaps of ILM, amniotic membrane, or other similar tissues. This step can be aided by iOCT thanks to its ability to show the surgeon the correct placement of these “tissue plugs” [32,33] (Figure 2).

Other than guiding the surgeon through the surgical procedure itself, OCT technology can be employed in order to predict postoperative outcomes. Indeed, with the use of iOCT, surgeons are able to analyze intraoperatively the amount of retinal stress and thickening induced by the peeling process. In a recent study by Runkle et al. [34], the inner retinal thickening immediately following peeling measured with iOCT was correlated with the postoperative development of a dissociated optic nerve fiber layer (DONFL), a finding consisting of numerous dark arcuate striae within the posterior pole in the direction of the optic nerve.

Since the introduction of OCT as routine investigation for macular holes, there have been numerous attempts to predict surgical success based on preoperative OCT parameters. Based on the results of previous studies, only preoperative minimal width of the macular hole appeared consistently as a robust predictor of hole closure [35,36]. However, since the morphology of macular holes significantly changes after peeling, iOCT variables indicating how the retinal layers responded to the surgical manipulation may help to predict macular hole closure. As reported by Ehlers et al. [37,38], eyes that underwent more significant reductions in minimum hole width and hole volume at the end of surgery exhibited a significantly higher closure rate at follow-up. These findings were interpreted by the author as intraoperative signs of higher tissue laxity that could justify the better anatomical results. 

Moreover, in a recent report by Nishitsuka and colleagues, hole diameter was measured through iOCT at different surgical stages, showing a decrease after ILM peeling and a further reduction after gas tamponading, probably due to the centripetal surface tension force between the retina and the gas bubble [39]. The ability of iOCT to observe the macular hole changes intraoperatively may also affect the recommendations for postoperative face-down positioning protocols. Typically, a face-down positioning of 3–4 days is recommended. However, as shown by Lorusso and colleagues [40], a shorter face-down positioning time of 12–24 h is equally effective when intraoperative macular hole closure can be demonstrated by iOCT.

Along with these positive prognostic factors, a study by Inoue et al. [41] found through iOCT that residual fragments of ILM/ERM at the end of peeling were negatively correlated with visual recovery after hole closure, probably due to a hampered healing process of the outer retina caused by these remnants. In contrast with their results, a recent report by Tao and colleagues [42] found tissue remnants at the hole’s edge to improve the closure rate. In their study, eyes with vertical pillars of tissue projected into the vitreous cavity (so-called “hole-door sign”) or horizontal flaps adherent to the hole edge (so-called “foveal flap”) were shown to have significantly better final visual acuity and postoperative restoration of the external limiting membrane compared to the eyes that had free edges. Thus, the role of residual tissue remains controversial and would benefit from further investigation.

### 3.3. Clinical Applications in Vitreoretinal Surgery: Retinal Detachment

Despite the recent technological and surgical advancements, rhegmatogenous retinal detachment (RRD) surgery remains a challenging procedure with elusive long-term functional results even after successful anatomical repair. Since the advent of OCT, the high-resolution view of retinal layers has provided useful feedback for surgical planning and prognostication, with imaging studies that have provided novel ultrastructural biomarkers such as ellipsoid zone disruption, cystoid degeneration, and outer retinal corrugations [43].

With regards to the intraoperative use, the DISCOVER study reported that iOCT provided surgeons with feedback deemed relevant in 36% of cases and altered the decision-making process in 12% of cases [15]. Additionally, although previously employed mainly in the analysis of the posterior pole, the intraoperative application of OCT technology has made the retinal periphery more accessible. Our group recently described the use of an intraocular 23-gauge side-scanning probe that provides a wide field of view of the retina, with the added ability to overcome anterior segment opacities and highlight peripheral residual tractional membranes or retinal breaks that could otherwise be neglected [44,45]. Nishitsuka and colleagues [46] analyzed with iOCT 50 cases of RRD surgery and identified peripheral cystoid degeneration around retinal holes and in the detached area in 54% of eyes. Although considered an innocuous lesion, the authors suggested a possible role of peripheral cystoid degeneration in the pathogenesis of RRD and regarded iOCT as a useful technique to further investigate this association. 

**Figure 3:**
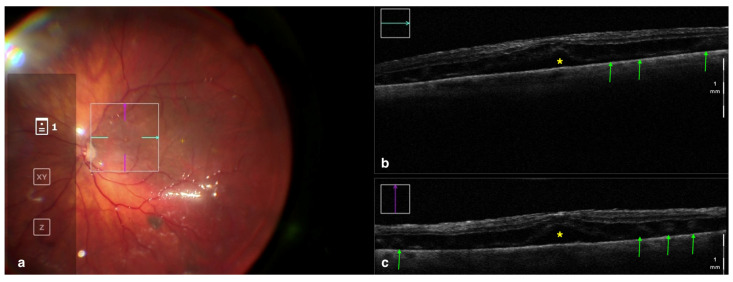
Rescan 700 iOCT showing a case of RRD after perfluorocarbon flattening. On the left is the en face ophthalmoscopic surgical view with a digital overlay indicating the scanning area (**a**) and in the upper and lower right portions of the image are the horizontal and vertical scans, respectively (**b**,**c**). The iOCT shows the surgeon the successful reattachment of the retina, although with persistence of subfoveal fluid (yellow asterisk) and the presence of outer retinal folds (green arrows) Abbreviations: iOCT, intraoperative optical coherence tomography; RRD, rhegmatogenous retinal detachment.

Besides better visualization of the retinal periphery, iOCT has made possible the real-time visualization of the subretinal space during the different steps of retinal reattachment surgery (Figure 3). Various studies [47,48,49] employed this technology intraoperatively and focused on the visualization of subretinal fluid. After pars plana vitrectomy and perfluorocarbon flattening of the retina, even when clinically inapparent, a variable amount of residual subfoveal fluid was identified with iOCT in all eyes and increased at the end of the surgery after fluid–air exchange. However, the fluid resorbed in all cases postoperatively, and no statistical correlation was found between the amount of fluid and long-term visual recovery [47,48,49]. 

With regards to ab externo RRD surgery, previous studies showed how scleral buckling and encircling procedures could also benefit from the application of this technology. In particular, Sotani et al. [50] reported how iOCT showed a mismatch between the buckle’s position and the retinal break in 5 eyes out of 12 cases, therefore helping the surgeons adjust the placing of the band and reach anatomical success in 100% of cases. 

### 3.4. Clinical Applications in Vitreoretinal Surgery: Submacular Surgery

Submacular surgery is a subfield of vitreoretinal surgery that was first introduced in the 1990s, when the first attempts to treat choroidal neovascularization and repopulate the subretinal space with healthy retinal pigment epithelium cells were described [51,52]. Currently, with the advent of anti-vascular endothelial growth factor drugs, submacular surgery has found new therapeutic indications such as the subretinal injection of therapeutical substances and the positioning under the retina of retinal grafts or prostheses.

Delivering drugs directly between photoreceptors and the retinal pigment epithelium allows one to reduce drug concentration. To do so, a 23- or 25-G cannula with a retractable 38- to 41-G tip is attached to a syringe controlled with the foot pedal of a vitrectomy machine [53]. This delicate procedure is associated with vision-threatening complications such as vitreous or choroidal hemorrhage, iatrogenic RRD or schisis, and reflux of the injected substance into the vitreous, which could potentially be limited with the visual feedback provided by iOCT. In 2022, Cehajic-Kapetanovic et al. tested the feasibility of iOCT for submacular injections. In their study, six patients underwent iOCT-guided robot-assisted subretinal injection of up to 100 µL of recombinant tissue plasminogen activator. The procedure was uneventful for all the participants, and there was no difference in the duration of surgery and tolerability between the robotic group and the manual surgery control group [54].

Another indication for subretinal injections is represented by inherited retinal diseases, a heterogeneous group of diseases primarily affecting the retinal pigment epithelium and photoreceptors. In order to treat genetic mutations, the preferred approach is the subretinal delivery of a viral vector, usually an adeno-associated virus or lentivirus. Currently, the only approved treatment for inherited retinal diseases is voretigene neparvovec-rzyl, an adeno-associated virus-mediated gene employed when treating certain forms of Leber’s congenital amaurosis and retinitis pigmentosa where the gene RPE65 is defective. As described by Hussain et al. [55] and Vasconcelos and colleagues [56], iOCT is an useful tool in confirming the correct positioning of the cannula in the subretinal space and the successful creation of the bleb with minimal overstretching of the retina. 

**Figure 4:**
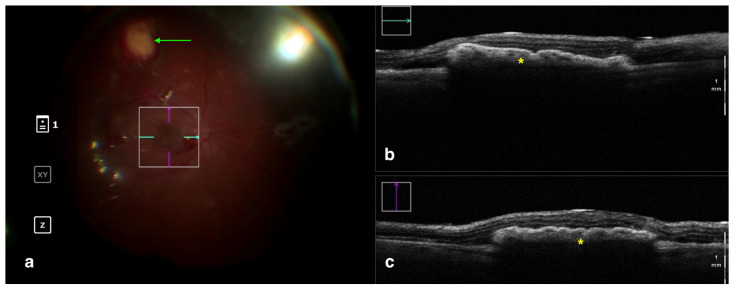
Rescan 700 iOCT showing a case of RPE transplantation. On the left is the en face ophthalmoscopic surgical view with a digital overlay indicating the scanning area (**a**). The green arrow indicates the sites used for harvesting the graft. In the upper and lower right portions of the image are the horizontal and vertical scans, respectively (**b**,**c**). The iOCT shows the surgeon the correct positioning and orientation of the graft under the retina (indicated by the yellow asterisk). Abbreviations: iOCT, intraoperative optical coherence tomography; RPE, retinal pigment epithelium.

Aside from guiding subretinal injection procedures, iOCT can also be useful for surgeries such as chorioretinal biopsies, retinal pigment epithelium transplantation, and retinal prosthesis implantation (Figure 4). In 2017, Browne et al. analyzed six eyes within the DISCOVER study that underwent chorioretinal biopsy under iOCT guidance. During the surgery, the authors benefited from real-time monitoring of the instrument–tissue interaction to facilitate the identification of the more informative and suitable biopsy sites, reach the proper biopsy position and depth, and verify retinal attachment and wound margin integrity after completion of the biopsy [57]. Similarly, in the same year, Grewal and colleagues described the case of a 46-year-old male with advanced retinitis pigmentosa who underwent implantation of an Argus II device under real-time feedback of microscope-integrated iOCT to guide the correct positioning and orientation of the prosthesis over the macula [58].

## 4. Conclusions: Current Limitations and Future Advancements 

Since the initial transfer of OCT devices from the clinic to the operating room, iOCT technology has greatly evolved, becoming a useful adjunct to ophthalmic surgery. However, some technical limitations still hinder the widespread adoption of this technology, and it remains unclear whether the significant investment and learning curve required to integrate this technology into the surgical workflow are justified by relevant clinical benefits for patients. 

From a technical point of view, there are still various critical points that have to be addressed, such as the shadowing effect cast by most of the surgical equipment commonly used currently in vitreoretinal surgery. To overcome this problem, OCT-compatible instruments composed of semitransparent rigid plastic material are under development [59,60].

Software-wise, the OCT devices available on the market are still unable to track and focus automatically on the areas of interest and are still dependent on the physical control of surgeons. The development of voice-activated controls or automatic features of tracking and focusing may allow a more fluid integration of OCT imaging into the surgical procedure. Moreover, the advent of high-level graphics processing units might, in future, allow one to acquire and render volumetric data in real time, thus providing three-dimensional surgical images instead of cross-sectional OCT scans [60,61].

Another promising area of research is the potential integration of iOCT in robotic-assisted surgical platforms. The Preceyes Surgical System (PSS; Preceyes B.V.) is a telemanipulated robotic device with an instrument-integrated iOCT sensor. Mounted near the tip of the instruments, the sensor allows micrometer-scale precision on anteroposterior movement thanks to an A-scan-based feedback loop with superhuman reactivity that keeps the instruments’ tip within safe virtual boundaries from retinal structures, reaching levels of efficacy and safety unmatchable by human dexterity [62].

In conclusion, iOCT represents a remarkable advancement for surgical visualization and decision making during vitreoretinal surgery. Integration of intraoperative OCT into routine surgical workflows, the cost-effectiveness of implementation, and the learning curve for surgeons are all factors that require careful consideration. However, as this technology continues to evolve, iOCT holds promise for enhanced surgical safety and precision, advancing the boundaries of minimally invasive surgery and ultimately leading to better patient outcomes. 

## Figures and Tables

**Table 1:** Commercially available microscope-integrated intraoperative OCT systems.

	RESCAN 700	EnFocus Ultra-HD	iOCT
Producer	Carl Zeiss Meditec	Leica Microsystems	Haag Streit Surgical
OCT Technology	Spectral Domain	Spectral Domain	Spectral Domain
Wavelength (nm)	840	860	840
Scan Speed (A-Scan/s)	27.000	32.000	10.000
Scan Depth (mm)	2	2.5	4.2
Axial Resolution (µm)	5.5	4.0	5.0

## Data Availability

Not applicable.
